# Supplementary material for: Contrasting Paternal and Maternal Genetic Histories of Thai and Lao Populations
Source: Mol Biol Evol. 2019 Apr 12;36(7):1490–506. doi: 10.1093/molbev/msz083 (PMC6573475; doi:10.1093/molbev/msz083)
Supplement: Supplement_Material_msz083 [file supplement_material_msz083.zip › Supplementary Material-final.pdf]

Supplementary Material

Contrasting paternal and maternal genetic histories of Thai and Lao populations

Wibhu Kutanan[1,2,*], Jatupol Kampuansai[3,4], Metawee Srikummool[5], Andrea Brunelli[6], Silvia Ghirotto[6], Leonardo Arias[2], Enrico Macholdt[2], Alexander Hübner[2], Roland Schröder[2], and Mark Stoneking[2,*]

This paper contains supplementary text, eight supplementary figures and seven supplementary tables.

Supplementary Text

Genetic relatedness among populations

The MA and MN show large differences from the other populations in the heat plots of Φ_{st} values (supplementary fig. 5). However, in general both MSY and mtDNA results show relatively larger genetic heterogeneity of the AA groups vs. genetic homogeneity of the TK and ST groups (supplementary fig. 3 and 5). After excluding these MA and MN as outliers, the first dimension of the plot divides the AA populations into two groups: one is diverged from the TK cloud, i.e. KH, KA, SU, TN, BU, BL, LW1 and LW3 and another is interspersed with the TK groups, i.e. SO, MO, BO, and PL (fig. 4A). The MDS heat plot for the MSY supported the divergence of AA populations and also emphasized the similarity between some AA populations and TK populations (fig. 4C). The ST speaking-Karen populations are close to the AA-speaking Mon in the right side of the plot (fig. 4A). Among the TK-speaking populations, BT2 and IS3 are closer to the AA groups on the left side of the plot while the central Thai (CT1-CT7) and one Khon Mueang group (KM7) are closer to the AA groups (MO, PL and LW2) and Karen on the right side (Fig. 4A), in agreement with the MDS heat plot for the MSY (fig. 4C). In the second dimension, the Lawa groups are very differentiated (fig. 4A), in accordance with the AMOVA (table 1) and heat plot results (fig. 4C). The heat plot of MSY Φ_{st} values supports strong genetic homogeneity in the TK and ST groups and also generally shows non-significant differences between the Mon (all groups) and the TK populations, especially with the CT groups, which are different from the other AA speaking populations (fig. 4C). For the MDS of mtDNA (fig. 4B), the Mon generally showed genetic affinity with the TK groups in the center of the plot, with the exception of MO1, MO5 and MO6, which differ from the other Mon groups, as can be also seen in the MDS plot (fig. 4B) and

heat plot (fig. 4D). Moreover, contrasting relationships based on the MSY vs. mtDNA was observed for the SK and SO from northeastern Thailand, and BT2 from central Thailand. The mtDNA differentiation from other populations was stronger than that for the MSY for the SK and SO, while opposite was observed for the BT2 (figs. 4A and 4B).

Thai MSY haplogroup distribution

Among the 928 MSY sequences from Thailand, there are 92 specific haplogroups. Because some of these are subhaplogroups of other haplogroups, we use the following nomenclature: an asterisk denotes a parent haplogroup and all subhaplogroups, while the lack of an asterisk denotes just that specific haplogroup. O1b* is by far the most frequent haplogroup (51.19%) and is present in all populations except the MN, who have only haplogroup K (fig. 1 and supplementary table 2). There are two subclades of O1b*: O1b1a1* or O-PK4* (99.37%), and O1b1a2 or O-Page59 (0.63%). Only O1b1a1* was previously reported to be wide spread in northern Thailand (Brunelli et al. 2017), while O1b1a2 is newly reported here, occurring in CT5, YU2 and PU4 (supplementary table 2). There are several subclades of O1b1a1*; the most frequent (50.54%) is O1b1a1a* or O-M95*, which occurs in almost all populations. However, almost half of the AA groups show a very high frequency of O-M95* (>70%), i.e. KH1-KH2, KA, BU, BL, SU, TN1-TN3, MA and LW3 (fig. 1 and supplementary table 2) while only two TK populations, i.e. BT2 (94.44%) and IS3 (72.22%) have a high frequency of O-M95*. It appears that the frequency of O-M95* is a major driver of the patterns in the MDS plot in dimension 1 (fig. 4A): we find that O-M95* is at high frequency in the populations on the left of the plot and gradually decreases to very low frequency in the populations on the right side, e.g. MO2, MO4, MO7, BO, CT4 and CT7 (fig. 4A).

O-M95* has also been reported to be frequent in AA populations from Cambodia (Zhang et al. 2015) and Laos (Cai et al. 2011), but infrequent in populations from southern China (Zhang et al. 2015) and rare elsewhere in MSEA (Trejaut et al. 2014). The CA analysis (based on haplogroup frequencies) also supports the divergence of these AA populations, with many O1b* sublineages, e.g. O1b1a1a1b1a (O-B426) and O1b1a1a1a1a* (O-F2758*) (supplementary fig. 1). With a total frequency at 11.10%, O1b1a1a1b1a* is prevalent in the LW3 (88.23%), BL (66.67%), LW1 (60.00%), KA (55.56%), and TN3 (47.06%) populations (supplementary table 2).

O2a* or O-M324* is the second most frequent haplogroup with an overall frequency of 25.86%; this haplogroup has relatively high frequency (>50%) in several populations: MO4 (53.85%), PL (66.67%), CT4 (55.55%), CT6 (55.55%), and KM7 (61.53%); and moderate frequencies in some Mon groups (MO5: 35.71%), some TK groups (KM9 (47.05%), LU3 (41.17%), and SH (44.44%), and all ST speaking Karen (KSK1 (41.67%), KSK2 (37.5%), KPA (36.36%)) (fig. 1 and supplementary table 2). Interestingly, we also observe a cline in O2a* frequency in the first dimension of the MDS plot that runs opposite to the O-M95* cline: O2a* is at higher frequency in populations located on the right of the plot and decreases in frequency toward the left side (fig. 4A).

Within O2a*, lineage O2a2b1a1a* or O-F8*, which is equivalent to O-M133*, is the most frequent (13.79% total frequency) and occurs in almost all populations, with fairly high frequencies in PL (50%), KM7 (46.15%), CT4 (38.88%), SH (38.88%), KSK2 (37.50%) and KPA (36.36%) (supplementary table 2). O-M133 has been reported in Han Chinese from Taiwan and Thai from Bangkok (12.73-21.59%) (Trejaut et al. 2014), Northern Han (11.36%), Southern Han (9.61%), Kinh (8.70%), Japanese (9.09%) and Dai (24.44%) (Poznik et al. 2016), but is very rare in Malaysia, Indonesia, the Philippines and South Asia (Trejaut et al. 2014; Poznik et al. 2016).

The last subhaplogroup of O observed in our study is O1a* or O-M119*. With a total frequency of 4.53%, O1a* is prevalent in three TK-speaking populations, i.e. LU2 (23.08%), PU4 (22.22%) and PT (22.22%) and occurs at low frequency in several populations, including central Thai (CT2-CT5), Laotian (LA1-LA2) and Lao Isan (IS1-IS3) (fig. 1 and supplementary table 2). O-M119* is thus spread across many TK-speaking groups, and also occurs at high frequency in Austronesian populations (Trejaut et al. 2014), indicating shared genetic lineages between TK and AN speaking groups. O-M119* occurs sporadically in just a few AA groups (Mon, SU and TN2) and at low frequency, in agreement with a previous study of Laos (Cai et al. 2011). The observed O-M119* sequences in the AA groups thus might reflect contact with TK groups.

Overall, the SEA-specific O1b* and O2a* haplogroups (with several sublineages) differentiate our studied populations into at least two main paternal sources, and the frequencies of these two haplogroups correspond to the major differentiation in the MDS plot (fig. 4A). However, there are also several minor non-SEA MSY lineages which promote divergence for some populations, e.g. the Lawa groups. Haplogroup N*, a sister clade of O*, is reported to be distributed in north Asian, Tibeto-Burman, and AA groups in southwestern China (Shi et al. 2013). Only one sublineage (N1c2b2 or N-L665) was found in this study (total frequency of 2.80%) and one third of N-L665 was restricted to LW2, enhancing the divergence of this population. It also sporadically occurs in some AA groups (MO2, MO6 and LW1), Karen (KSK1) and TK groups (THK, LU3, CT1, CT2, KM1, KM2 and LA1) (supplementary table 2).

We also observed some minor haplogroups that are abundant in South/Central Asia (Lippold et al. 2014; Karmin et al. 2015; Poznik et al. 2016), e.g. R*, H*, and J*, occurring at total frequencies of 4.18%, 1.40% and 1.62%, respectively (fig. 1). Haplogroup R* is observed in all Mon groups (41.46% of R) except for MO3, and is at highest frequency in MO2 (33.33%)

(supplementary table 2). The same proportion of this haplogroup (41.46% of R*) is also detected in all central Thai groups, except for CT2, and is at high frequency in CT3 (26.32%) and CT7 (27.78%), providing more support for genetic connections between Mon and Central Thais. The remaining proportion (17.02%) of R* was found sporadically (only single samples each) in KH1, SU, YU1, IS1, IS4, BT1 and PU1, probably reflecting recent admixture/gene flow. In agreement with these observations, the CA plots show a correspondence between R1a1a1b (R-Z647) and some central Thai and Mon groups (supplementary fig. 1). Haplogroup H* shows a similar haplogroup distribution, i.e. occurring in both some Mon (MO2-MO4 and MO7) and some central Thai (CT1 and CT3-CT5) groups. Haplogroup H* also occurs sporadically in the KH1, YU2, SH and KL groups. Elsewhere, haplogroup H* is found in Burmese and Malayan populations (Karmin et al. 2015) and Central Asian populations (Lippold et al. 2014). Haplogroup J2* is distributed in AA speaking Mon (MO2, MO5, MO6 and MO7), BO, BU, TN3 and TK speaking central Thai (CT5 and CT7) and Lao Isan (IS1 and IS2). Haplogroup J2* has been found in many populations from Central Asia (Lippold et al. 2014). Generally, the haplogroup profile indicates genetic affinities between the Mon and South/Central Asian groups, which is consistent with the MDS plots (fig. 4A) and results from mtDNA analyses (Kutanan et al. 2017; Kutanan et al. 2018b).

The other minor haplogroups observed in this study are D1*, G1b and F. Haplogroup D1* was found with the highest frequency in NY (33.33%) followed by KPA (27.27%), and occurs at lower frequency in a few other groups (supplementary table 2). Subclade D1a1a (D-N1) is prevalent in Tibetan groups and ST-speaking Qiang in southwestern China, and less prevalent in MSEA (Shi et al. 2008; Wang et al. 2013). Haplogroup G1b (G-L835/L830) was restricted to the Karen, where it was found in three of the four Karen groups. G1* was previously reported to occur in Southwest and Central Asia (Balanovsky et al. 2015). The CA analysis also supports the

divergence of the Karen (KSK1, KSK2 and KPW) based on G1b, the divergence of SK based on F, and the differentiation of NY and KPA based on D1a1a (supplementary fig. 1). In general, the occurrence of both SEA specific haplogroups and haplogroups prevalent in North Asia/Tibet and Southwest Asia in the Karen suggest multiple parental sources, in agreement with previous studies based on mtDNA (Kutanan et al. 2014; Kutanan et al. 2018b). Haplogroup F (F-M89) was distributed at low frequency in SO, SK and PU4, who migrated from Vietnam during historical times (Schliesinger 2000) and was also reported in one Kinh sample from Vietnam (Poznik et al. 2016) and five Lahu samples from southern China (Lippold et al. 2014). The origins of this haplogroup are uncertain but it might have originated in the area of present-day Vietnam and southern China; additional studies of Vietnamese populations would be informative.

References

Balanovsky O, Zhabagin M, Agdzhoyan A, Chukhryaeva M, Zaporozhchenko V, Utevska O, Highnam G, Sabitov Z, Greenspan E, Dibirova K, et al. 2015. Deep Phylogenetic Analysis of Haplogroup G1 Provides Estimates of SNP and STR Mutation Rates on the Human Y-Chromosome and Reveals Migrations of Iranic Speakers. PLoS ONE 10(4): e0122968.

Brunelli A, Kampuansai J, Seielstad M, Lomthaisong K, Kangwanpong D, Ghirotto S, Kutanan W. 2017. Y chromosomal evidence on the origin of northern Thai people. PLoS ONE 12(7): e0181935.

Cai X, Qin Z, Wen B, Xu S, Wang Y, Lu Y, Wei L, Wang C, Li S, Huang X, et al. 2011. Human Migration through Bottlenecks from Southeast Asia into East Asia during Last Glacial Maximum Revealed by Y Chromosomes. PLoS ONE 6(8): e24282.

Karmin M, Saag L, Vicente M, Wilson Sayres MA, Järve M, Talas UG, Rootsi S, Ilumäe AM, Mägi R, Mitt M, et al. 2015. A recent bottleneck of Y chromosome diversity coincides with a global change in culture. Genome Res. 25: 459–466.

Kutanan W, Srikummool M, Pittayaporn P, Seielstad M, Kangwanpong D, Kumar V, Prombanchachai T, Chantawannakul P. 2015. Admixed origin of the Kayah (Red Karen) in Northern Thailand Revealed by Biparental and Paternal Markers. Ann Hum Genet. 7: 108–122.

Kutanan W, Kampuansai J, Srikummool M, Kangwanpong D, Ghirotto S, Brunelli A, Stoneking M. 2017. Complete mitochondrial genomes of Thai and Lao populations indicate an ancient origin of Austroasiatic groups and demic diffusion in the spread of Tai–Kadai languages. Hum Genet. 136 (1): 85-98.

Kutanan W, Kampuansai J, Brunelli A, Ghirotto S, Pittayaporn P, Ruangchai S, Schröder R, Macholdt E, Srikummool M, Kangwanpong D, et al. 2018b. New insights from Thailand into the maternal genetic history of Mainland Southeast Asia. Eur J Hum Genet. 26(6): 898-911.

Lippold S, Xu H, Ko A, Li M, Renaud G, Butthof A, Schröder R, Stoneking M. 2014. Human paternal and maternal demographic histories: insights from high-resolution Y chromosome and mtDNA sequences. Investigative Genet. 5: 13.

Poznik GD, Xue Y, Mendez FL, Willems TF, Massaia A, Wilson Sayres MA, Ayub Q, McCarthy SA, Narechania A, Kashin S, et al. 2016. Punctuated bursts in human male demography inferred from 1,244 worldwide Y-chromosome sequences. Nat Genet. 48: 593–599.

Schliesinger J. 2000. Ethnic groups of Thailand: non-Tai-speaking peoples. Bangkok: White Lotus Press.

Shi H, Zhong H, Peng Y, Dong YL, Qi XB, Zhang F, Liu LF, Tan SJ, Ma RZ, Xiao CJ, et al. 2008. Y chromosome evidence of earliest modern human settlement in East Asia and multiple origins of Tibetan and Japanese populations. BMC Biol. 6: 45.

Shi H, Qi X, Zhong H, Peng Y, Zhang X, Ma RZ, Su B. 2013. Genetic Evidence of an East Asian Origin and Paleolithic Northward Migration of Y-chromosome Haplogroup N. PLoS ONE 8(6): e66102.

Trejaut JA, Poloni ES, Yen J-C, Lai YH, Loo JH, Lee CL, He CL, Lin M, et al. 2014. Taiwan Y-chromosomal DNA variation and its relationship with Island Southeast Asia. BMC Genet. 15: 77.

Wang C-C, Li H. 2013. Inferring human history in East Asia from Y chromosomes. Investigative Genet. 4: 11.

Supplementary figures

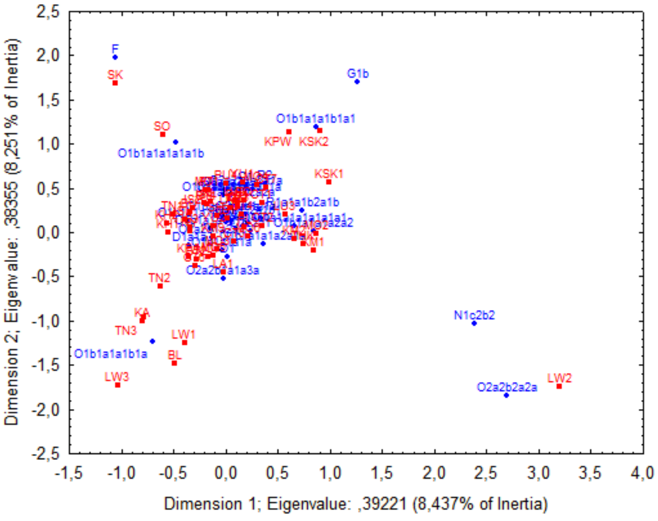

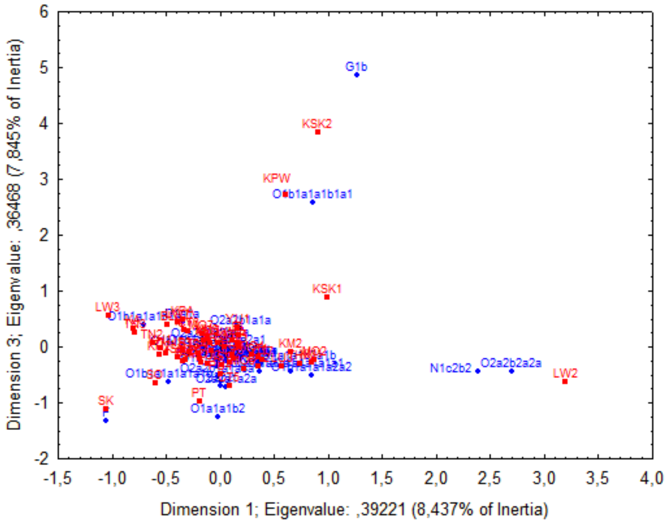

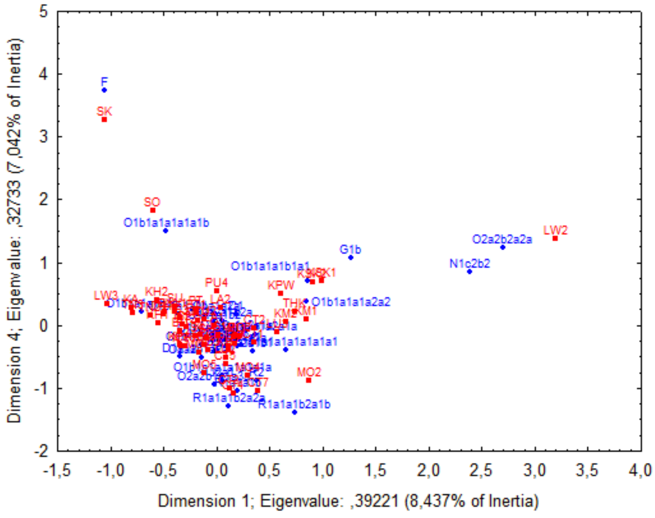

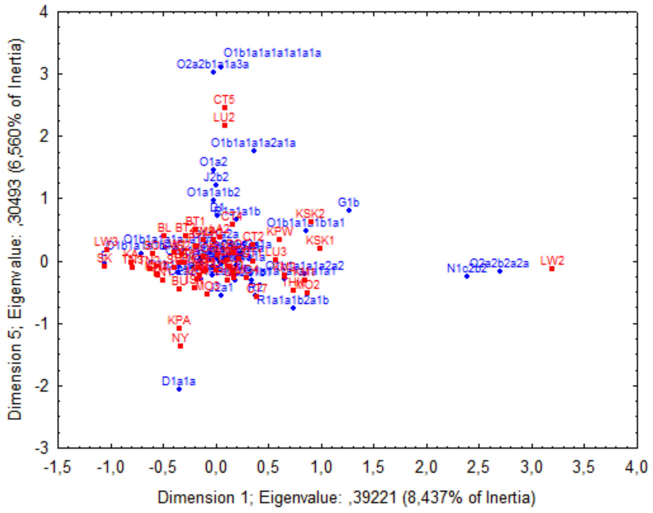

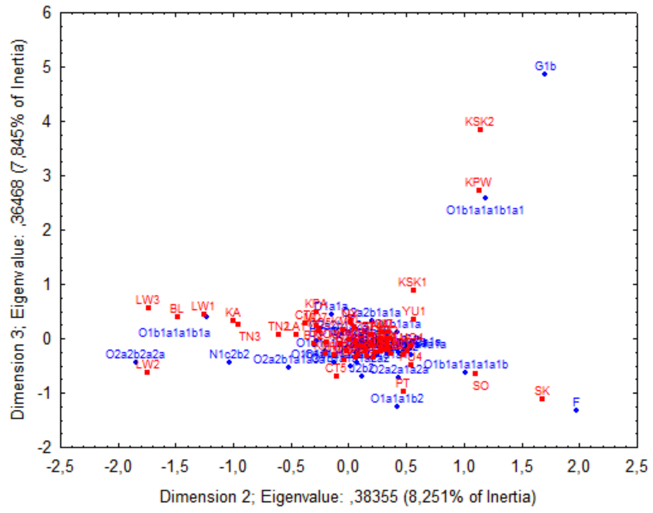

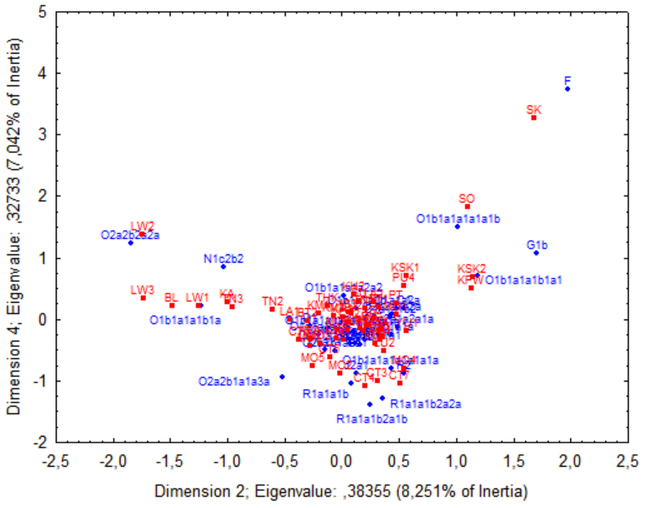

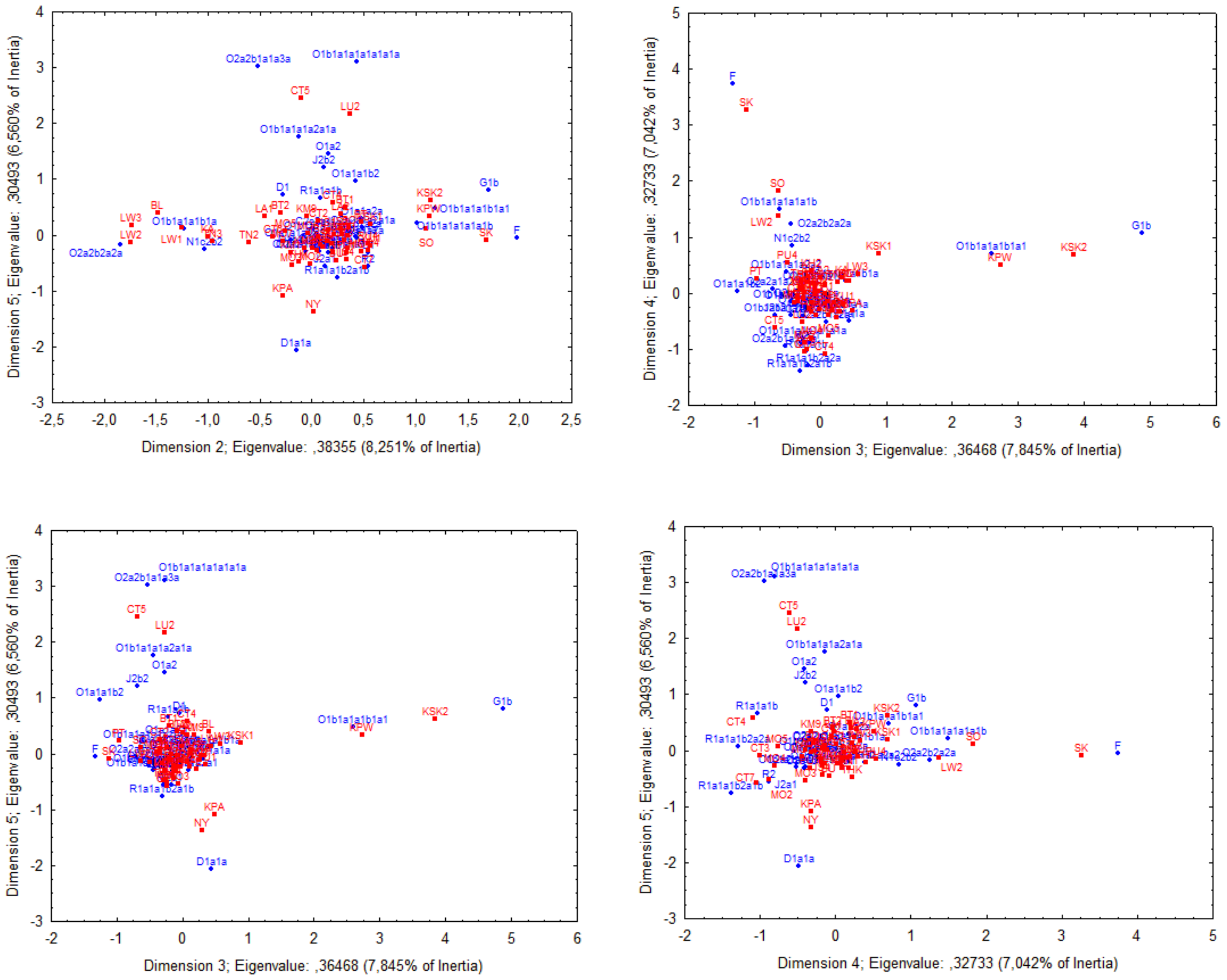

Supplementary fig. 1 Correspondence Analysis (CA) results based on haplogroup frequency of 58 populations excluding Maniq (MN). Population abbreviations are shown in fig. 1 and supplementary table 1.

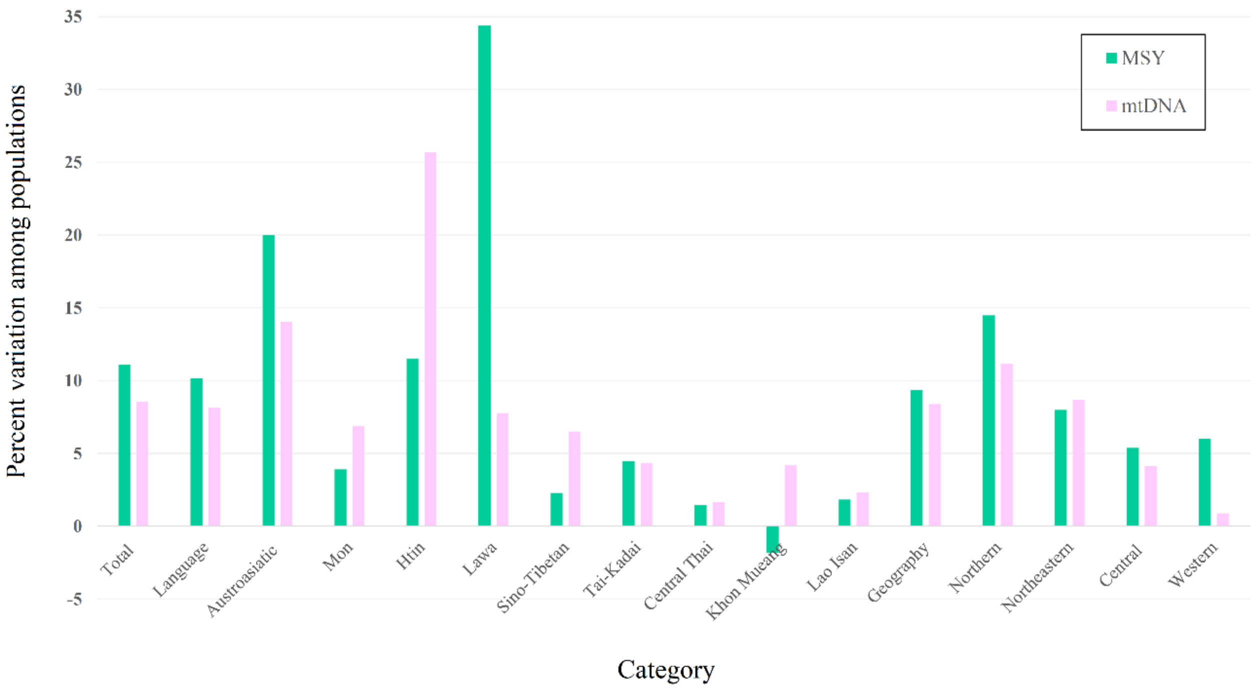

Supplementary fig. 2 Percent variation among populations in various linguistic or geographic categories, calculated by AMOVA.

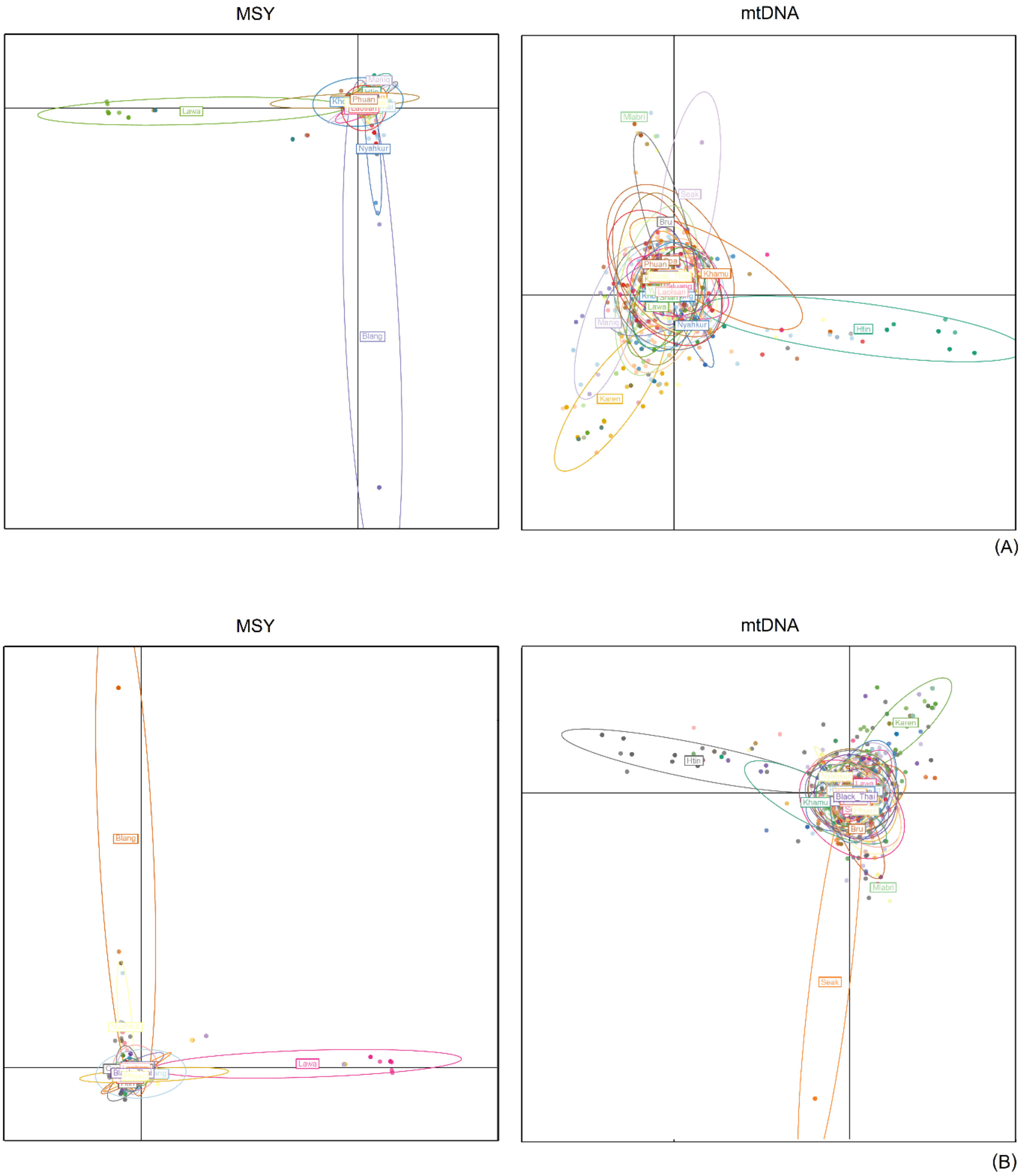
MSY
mtDNA
Lawa
Phuan
Nyahkur
Blang
Mlabri
Seak
Bru
Khamu
Htin
Karen
(A)
MSY
mtDNA
Blang
Lawa
Karen
Htin
Khamu
Black_Thai
Bru
Mlabri
Seak
(B)

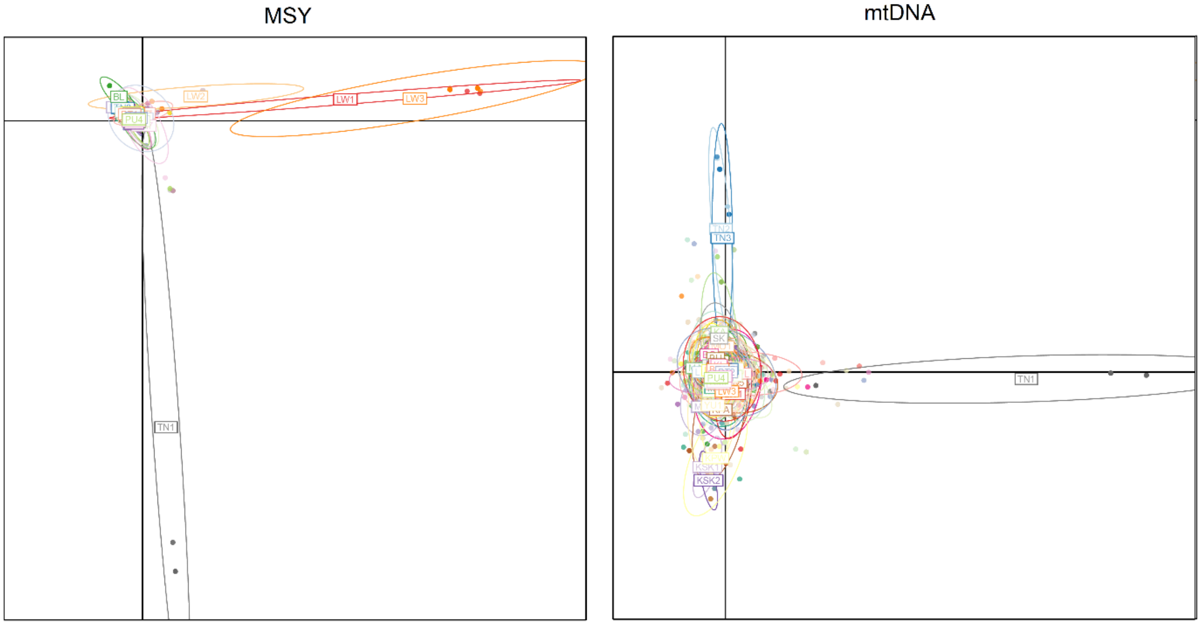

(C)

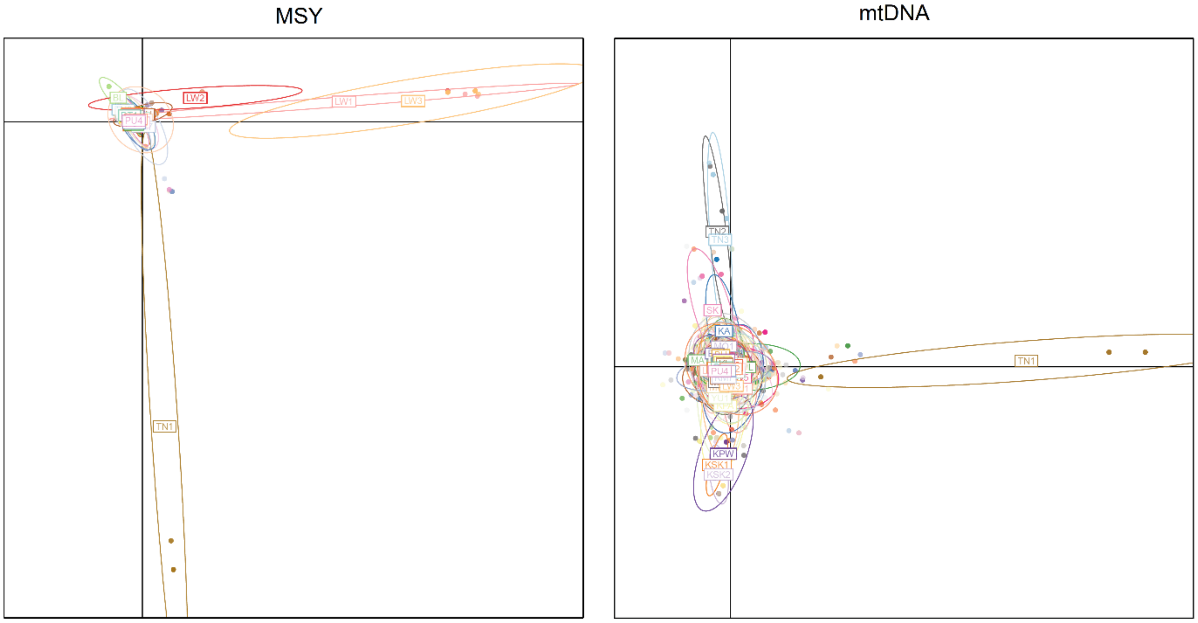

(D)

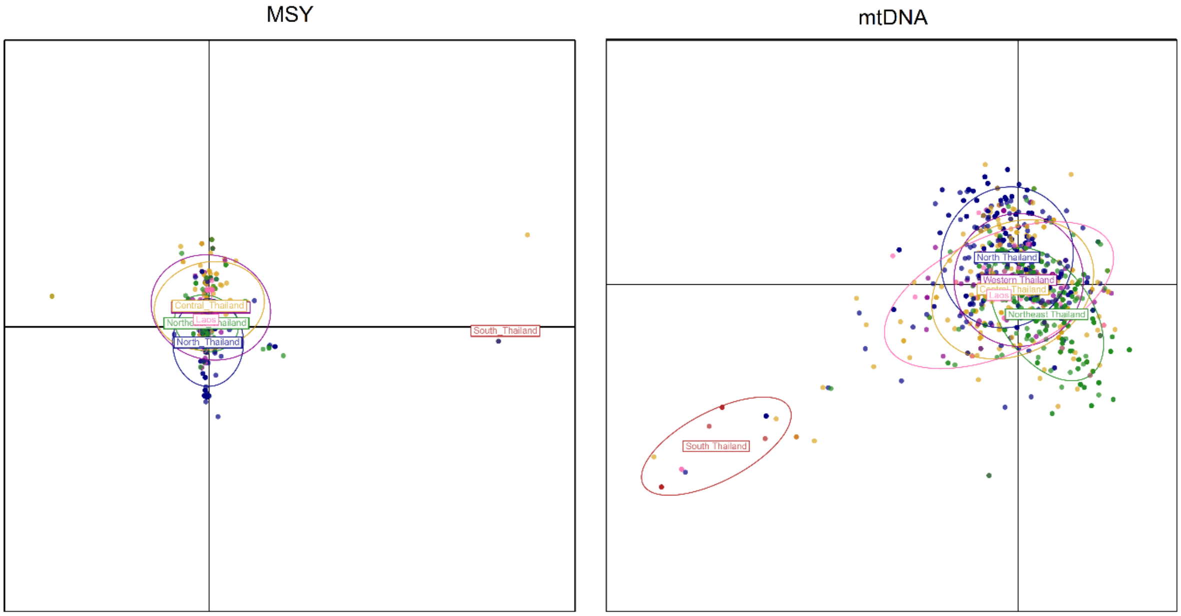
MSY
mtDNA
Central_Thailand
North_Thailand
South_Thailand
North Thailand
Western Thailand
Northeast Thailand
South Thailand

(E)

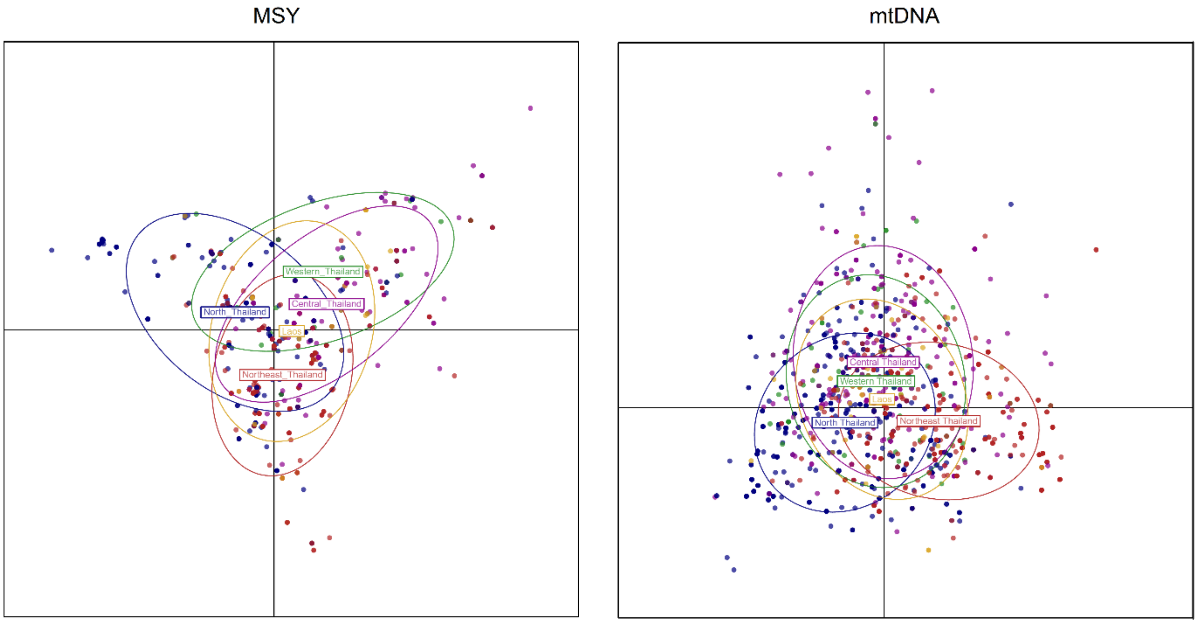
MSY
mtDNA
Western_Thailand
Central_Thailand
North_Thailand
Northeast_Thailand
Central Thailand
Western Thailand
North Thailand
Northeast Thailand

(F)

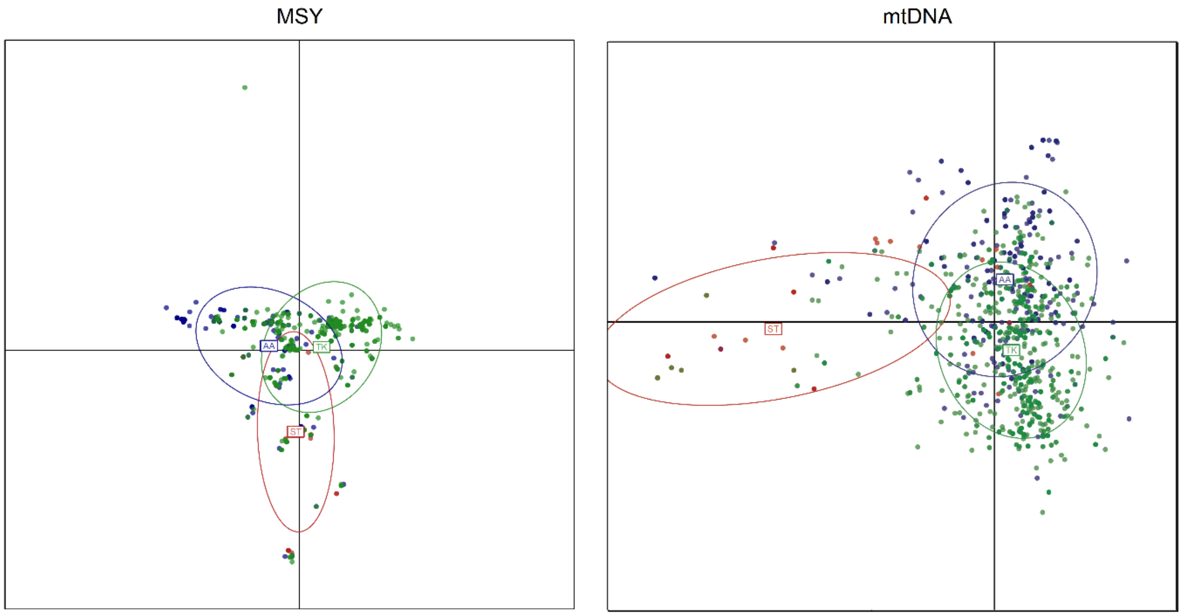

(G)

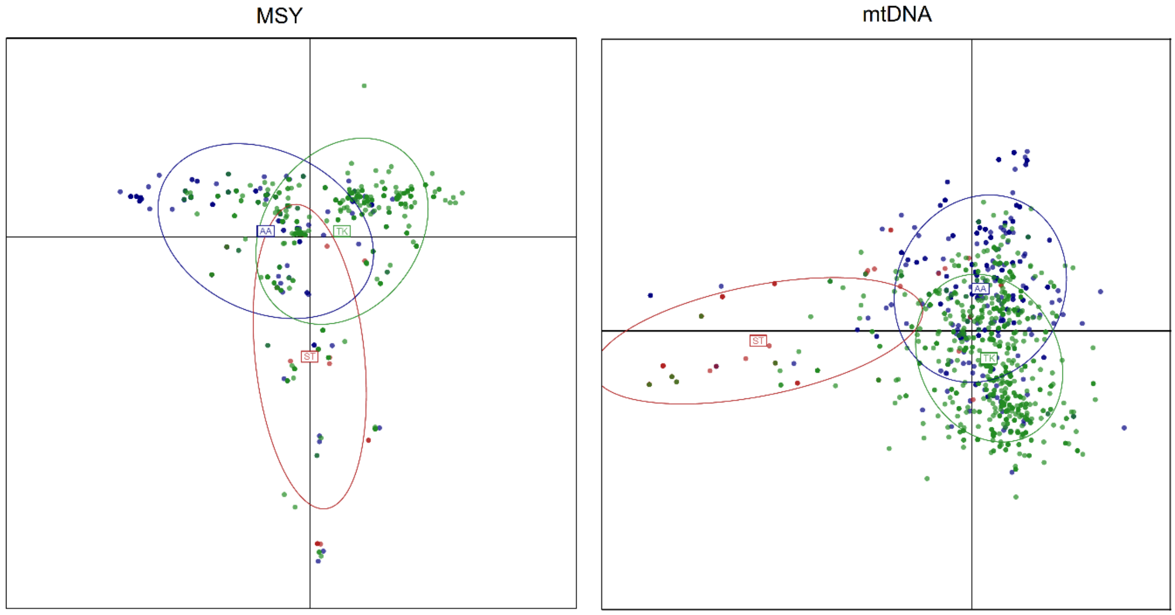

(H)

Supplementary fig. 3 The DAPC results based on ethnicity, population, geography and language (A, C, E and G, respectively). The DAPC results, excluding the Maniq based on ethnicity, population, geography and language (B, D, F and H).

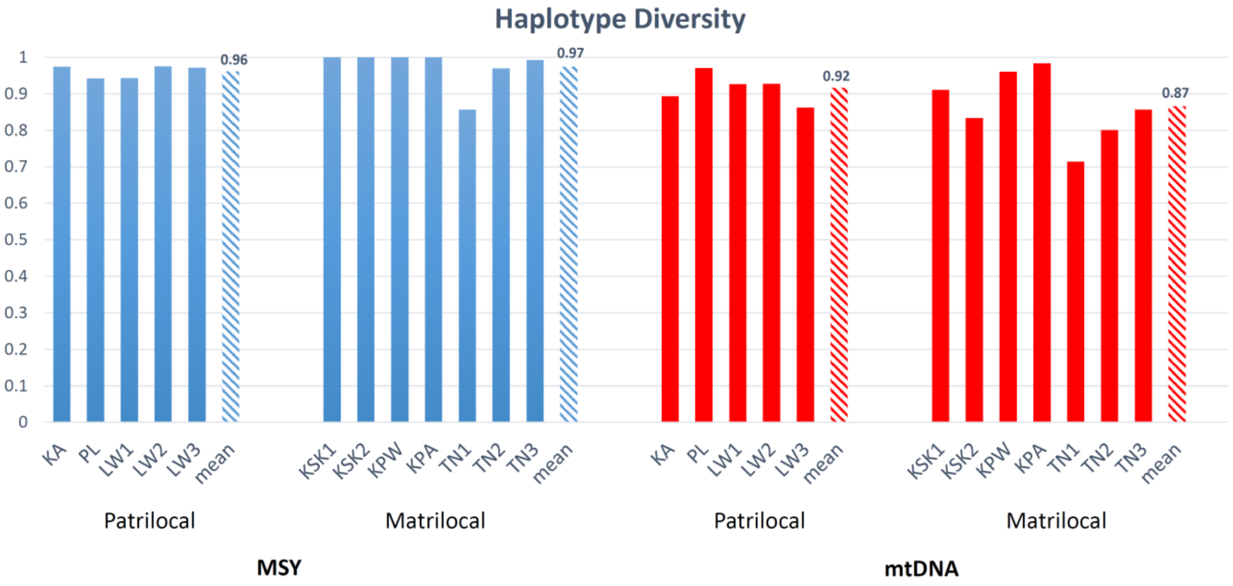

Haplotype Diversity
1
0.9
0.8
0.7
0.6
0.5
0.4
0.3
0.2
0.1
0
0.96
0.97
0.92
0.87
KA PL LW1 LW2 LW3 mean
KSK1 KSK2 KPW KPA TN1 TN2 TN3 mean
KA PL LW1 LW2 LW3 mean
KSK1 KSK2 KPW KPA TN1 TN2 TN3 mean
Patrilocal
Matrilocal
Patrilocal
Matrilocal
MSY
mtDNA

A

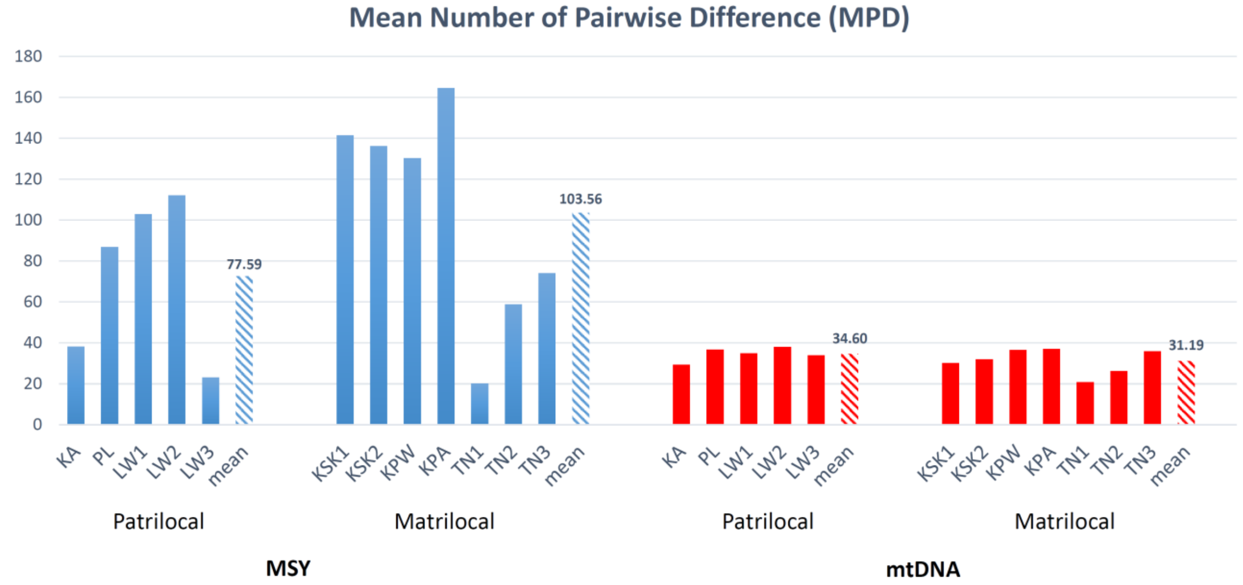

Mean Number of Pairwise Difference (MPD)
180
160
140
120
100
80
60
40
20
0
77.59
103.56
34.60
31.19
KA PL LW1 LW2 LW3 mean
KSK1 KSK2 KPW KPA TN1 TN2 TN3 mean
KA PL LW1 LW2 LW3 mean
KSK1 KSK2 KPW KPA TN1 TN2 TN3 mean
Patrilocal
Matrilocal
Patrilocal
Matrilocal
MSY
mtDNA

B

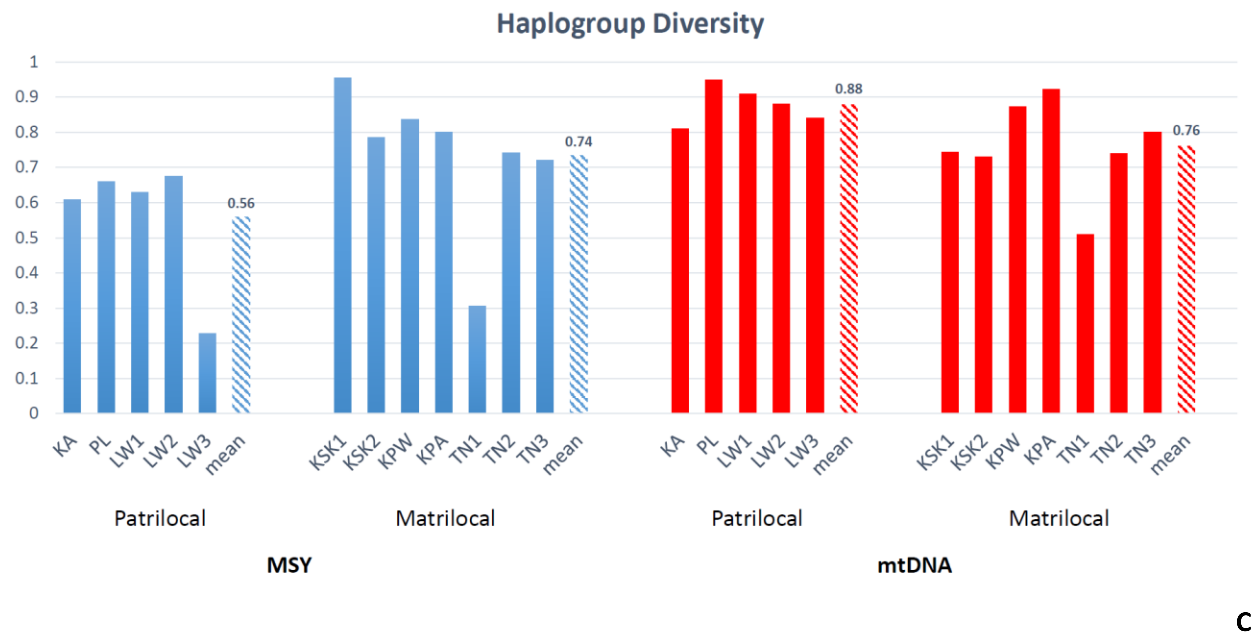

Supplementary fig. 4 The bar plot graphs of within population genetic variation values, i.e. haplotype diversity (A), MPD (B) and haplogroup diversity (C) in patrilocal and matrilocal groups. The shaded bar in each group indicates the mean diversity in each group.

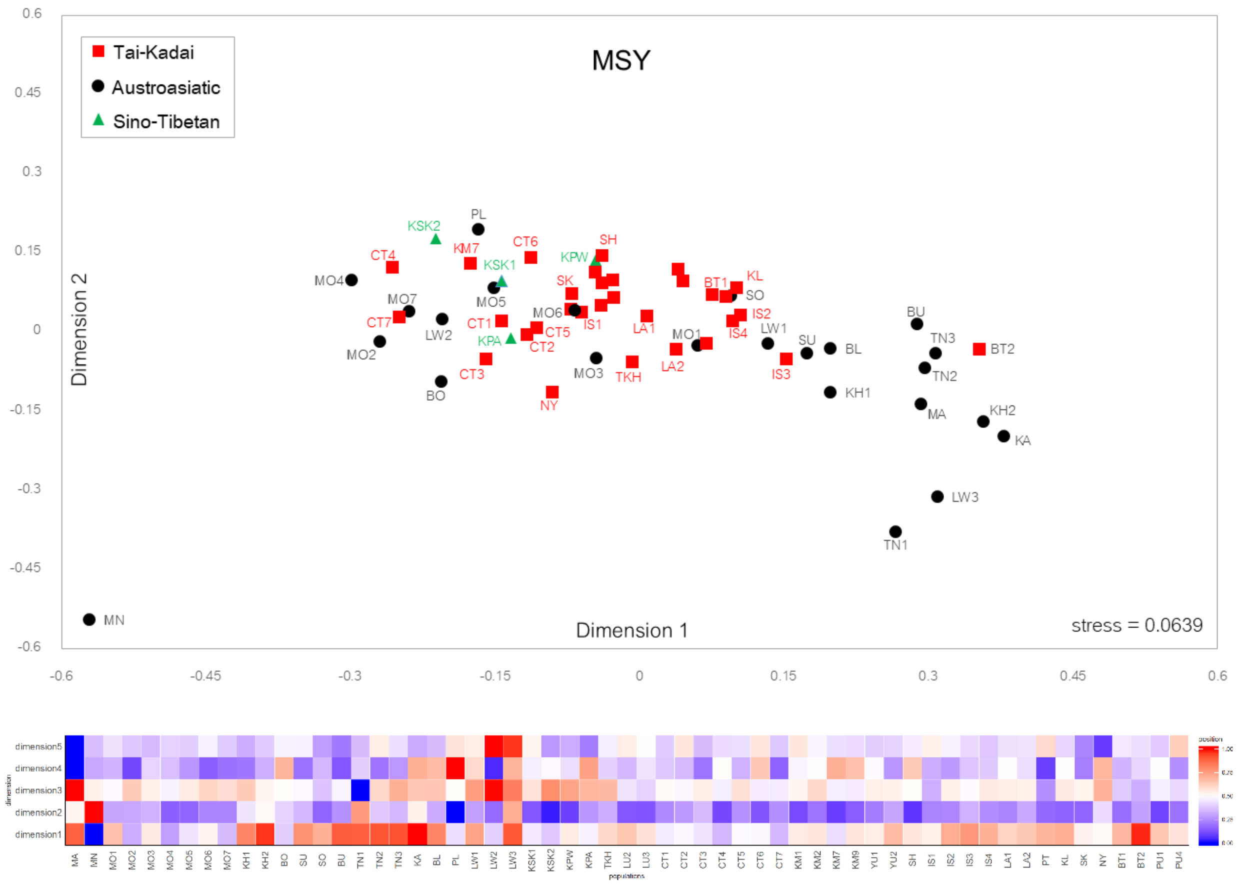
MSY
Tai-Kadai
Austroasiatic
Sino-Tibetan
Dimension 1
Dimension 2
stress = 0.0639

(A)

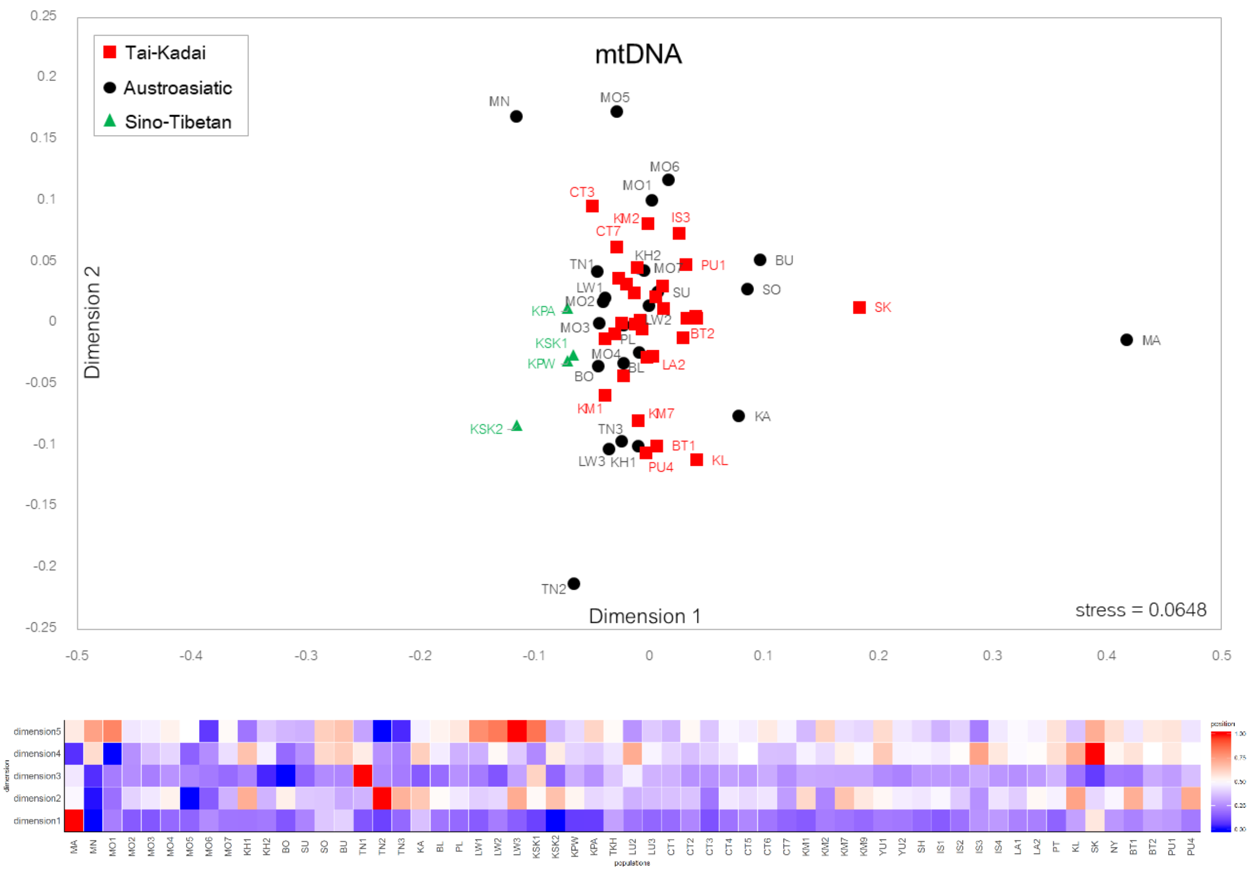

(B)

Supplementary fig. 5 The MDS plot and associated heat plot based on the Φ_{st} distance matrix calculated from the dataset for 59 populations, for the MSY (A) and mtDNA (B). Population abbreviations are in fig. 1 and supplementary table 1.

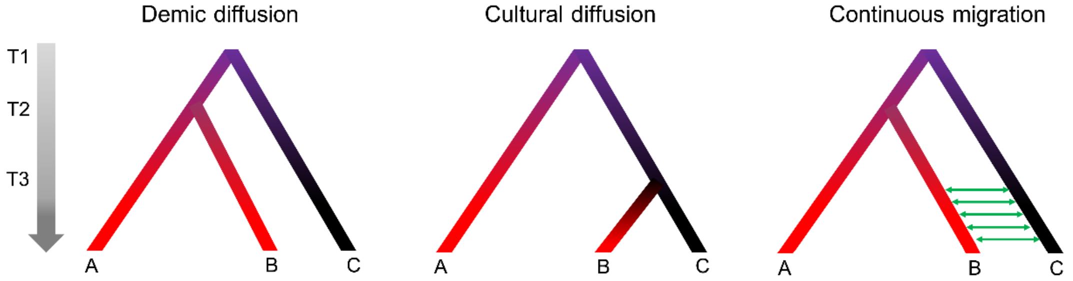

Model tests	A	B	C
Test 1	Xishuanbanna Dai	Khon Mueang	Lawa and Mon
Test 2	Xishuanbanna Dai	Lao Isan and Laotian	Suay, Khmu, Bru, Soa and Khmer
Test 3	Xishuanbanna Dai	Laotian	Suay, Khmu, Bru, Soa and Khmer
Test 4	Laotian	Lao Isan	Khmer
Test 5	Xishuanbanna Dai	Central Thais	Mon and Khmer

Supplementary fig. 6 Three demographic models for the ABC analysis (demic diffusion, cultural diffusion and continuous migration). A, B and C represent the different populations and Test 1-5 are the different datasets used in each test. T1, T2 and T3 are either divergence time or time of gene flow.

Test 1

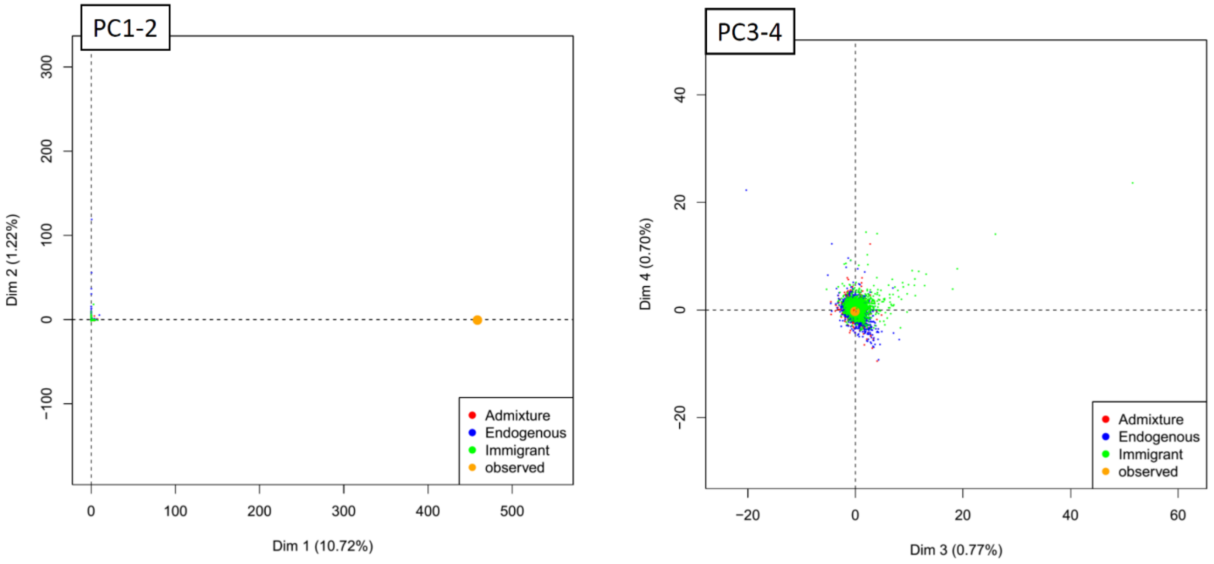
PC1-2
Dim 1 (10.72%)
Dim 2 (1.22%)
0
100
200
300
400
500
−100
Admixture
Endogenous
Immigrant
observed
PC3-4
Dim 3 (0.77%)
Dim 4 (0.70%)
−20
0
20
40
60
Admixture
Endogenous
Immigrant
observed

Test 2

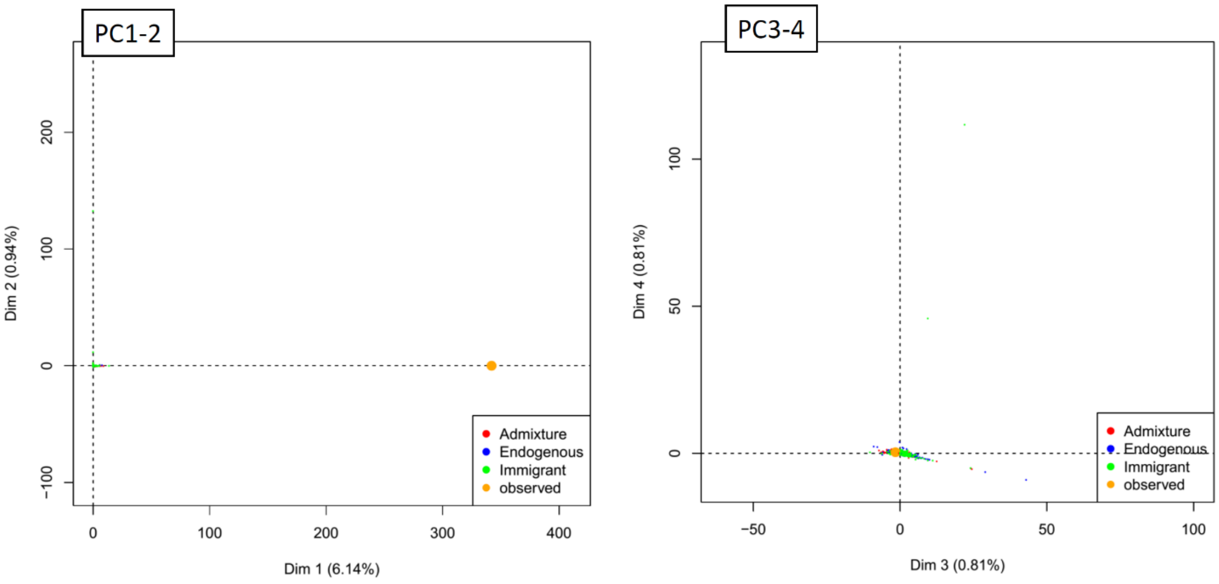
PC1-2
Dim 1 (6.14%)
Dim 2 (0.94%)
0
100
200
300
400
−100
Admixture
Endogenous
Immigrant
observed
PC3-4
Dim 3 (0.81%)
Dim 4 (0.81%)
−50
0
50
100
Admixture
Endogenous
Immigrant
observed

Test3

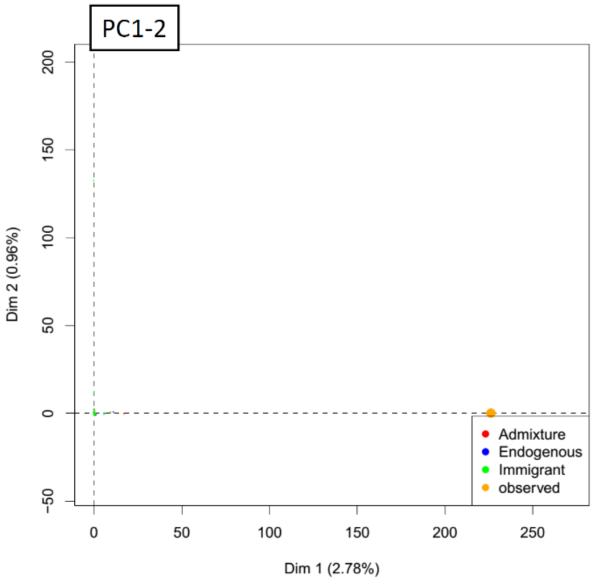
PC1-2
Dim 1 (2.78%)
Dim 2 (0.96%)
Admixture
Endogenous
Immigrant
observed

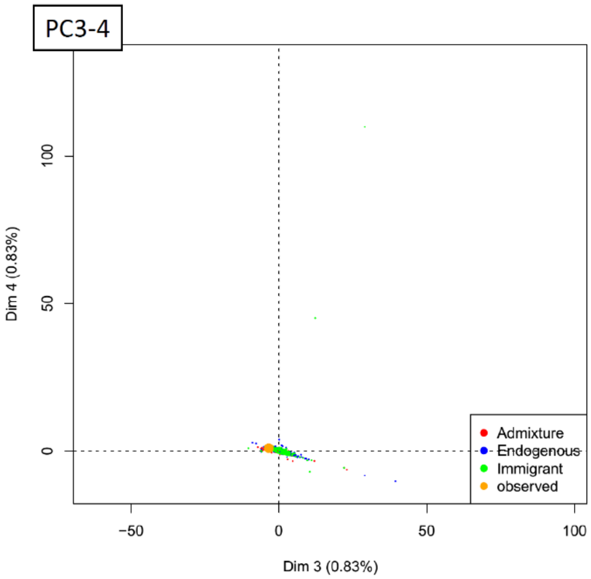
PC3-4
Dim 3 (0.83%)
Dim 4 (0.83%)
Admixture
Endogenous
Immigrant
observed

Test 4

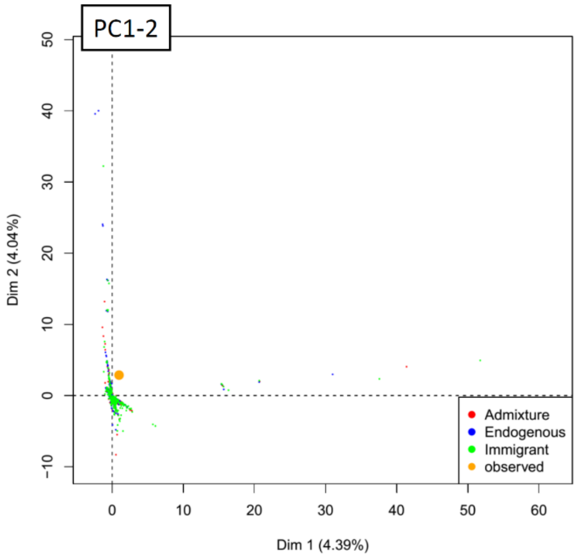
PC1-2
Dim 1 (4.39%)
Dim 2 (4.04%)
Admixture
Endogenous
Immigrant
observed

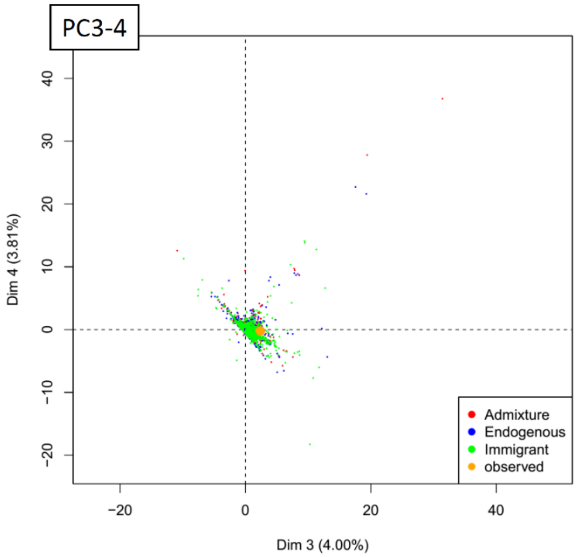
PC3-4
Dim 3 (4.00%)
Dim 4 (3.81%)
Admixture
Endogenous
Immigrant
observed

Test 5

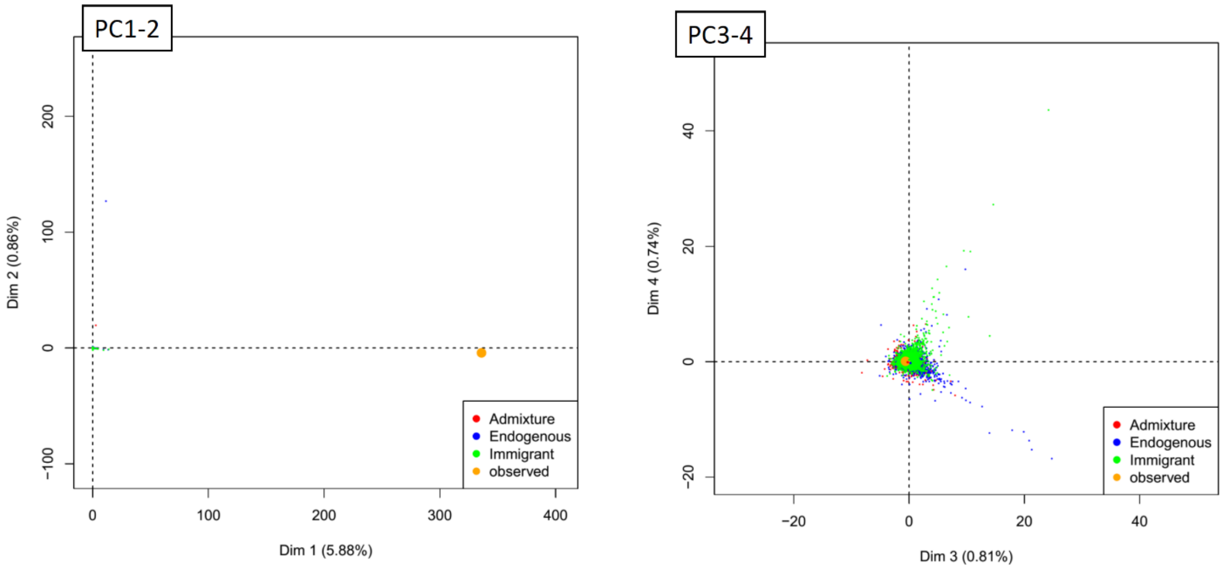

Supplementary fig. 7 PCA (Principle Component Analysis) analysis based on Dimension 1 and 2 and Dimension 3 and 4 for the fit between the observed data and the simulated data generated by each model for the origin of Northern Thai (Test 1), Laotian and Lao Isan (Test 2), Laotian (Test 3), Lao Isan (Test 4) and Central Thais (Test 5).

Test 1

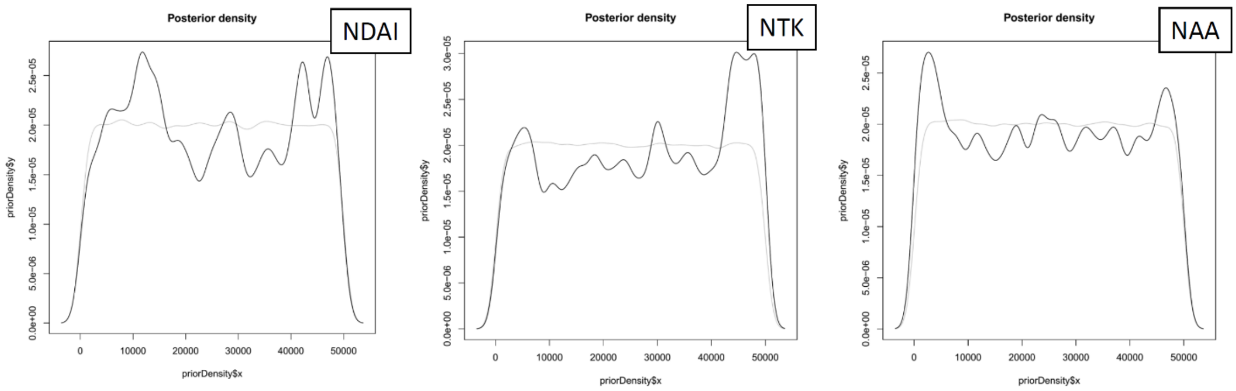

Test 2

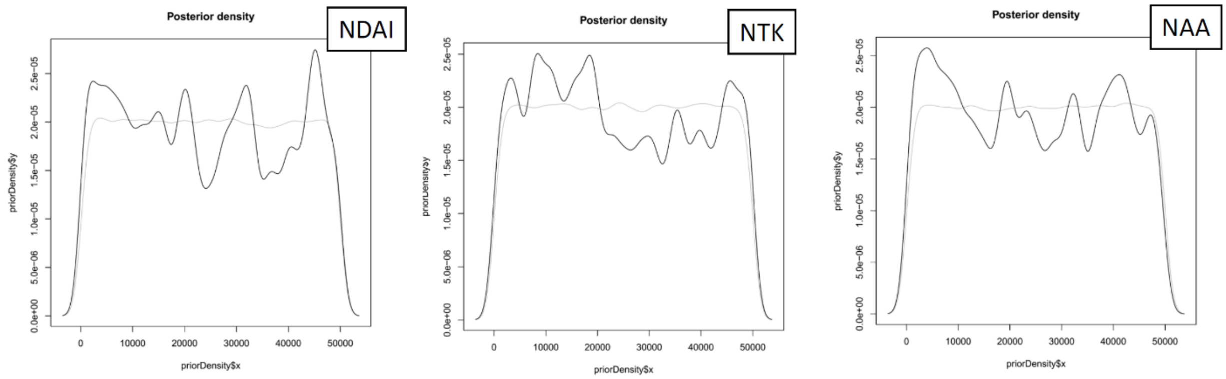

Test 3

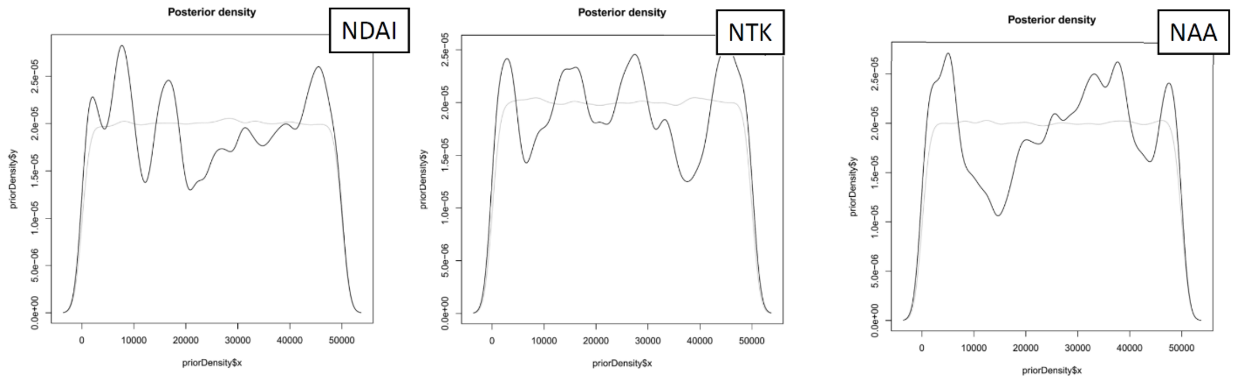

Test 4

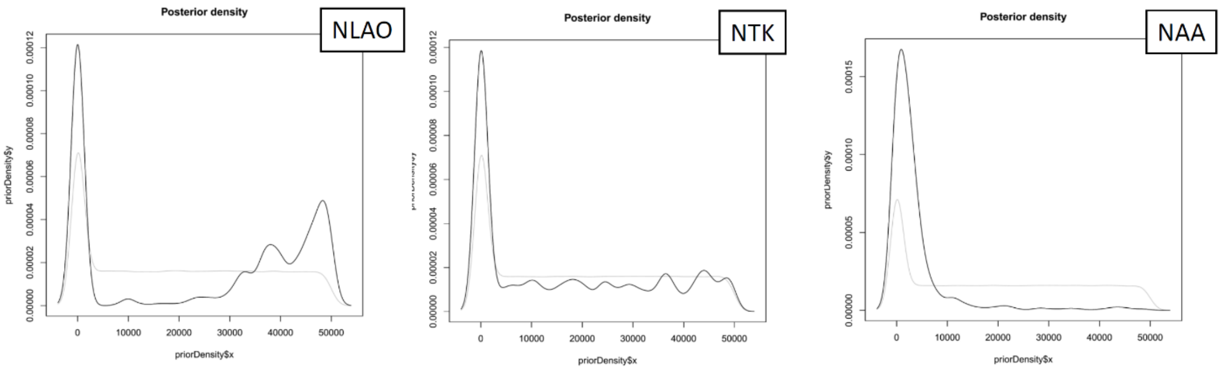

Test 5

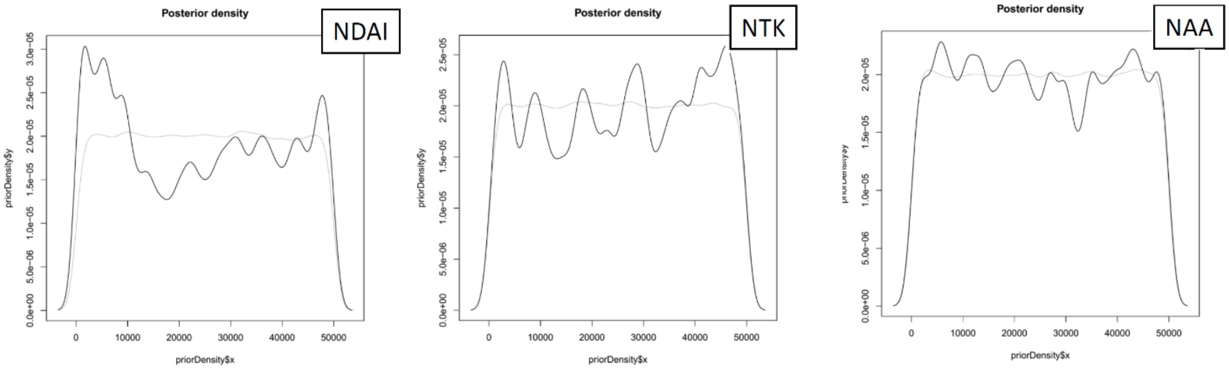

Supplementary fig. 8 Graphs representing the posterior distribution of each estimated effective population sizes over the extent of the prior range (solid black) and the prior distribution of each parameter (light gray) in each model for the origin of Northern Thai (Test 1), Laotian and Lao Isan (Test 2), Laotian (Test 3), Lao Isan (Test 4) and Central Thais (Test 5).

Supplementary table (excel file)

**Supplementary table 1**	General information and genetic diversity values of the studied populations.
**Supplementary table 2**	Haplogroup frequency.
**Supplementary table 3**	Votes assigned to each model by the Random Forest procedure and posterior probability for the selected model in the ABC analysis.
**Supplementary table 4**	Parameters estimation for the selected model in each ABC analysis tested.
**Supplementary table 5**	Genetic differences (Φst and corrected pairwise differences) between each groups of population used in ABC testes. Numbers in parentheses indicate *P*-values.
**Supplementary table 6**	MSY probe set details.
**Supplementary table 7**	Details for the compared populations for MSY data.
